# Supplementary material for: A Critical View of the Application of the APEX Software (Aqueous Photochemistry of Environmentally-Occurring Xenobiotics) to Predict Photoreaction Kinetics in Surface Freshwaters
Source: Molecules. 2019 Dec 18;25(1):9. doi: 10.3390/molecules25010009 (PMC7017383; doi:10.3390/molecules25010009)
Supplement: Supplementary file 1 [file molecules-25-00009-s001.zip › 3.2.4_gcc-4.4.0.zip!/3.2.4_gcc-4.4.0/3.2.4_gcc-4.4.0/doc/octave/html/interpreter/Integer-Conversions.html]

Integer Conversions - Untitled


Next: Floating-Point Conversions,
Previous: Table of Output Conversions,
Up: C-Style I/O Functions


---

#### 14.2.8 Integer Conversions

This section describes the options for the %d, %i,
%o, %u, %x, and %X conversion
specifications. These conversions print integers in various formats.

The %d and %i conversion specifications both print an
numeric argument as a signed decimal number; while %o,
%u, and %x print the argument as an unsigned octal,
decimal, or hexadecimal number (respectively). The %X conversion
specification is just like %x except that it uses the characters
ABCDEF as digits instead of abcdef.

The following flags are meaningful:

-: Left-justify the result in the field (instead of the normal right-justification). +: For the signed %d and %i conversions, print a plus sign if the value is positive. : For the signed %d and %i conversions, if the result doesn't start with a plus or minus sign, prefix it with a space character instead. Since the + flag ensures that the result includes a sign, this flag is ignored if you supply both of them. #: For the %o conversion, this forces the leading digit to be 0, as if by increasing the precision. For %x or %X, this prefixes a leading 0x or 0X (respectively) to the result. This doesn't do anything useful for the %d, %i, or %u conversions. 0: Pad the field with zeros instead of spaces. The zeros are placed after any indication of sign or base. This flag is ignored if the - flag is also specified, or if a precision is specified.

If a precision is supplied, it specifies the minimum number of digits to
appear; leading zeros are produced if necessary. If you don't specify a
precision, the number is printed with as many digits as it needs. If
you convert a value of zero with an explicit precision of zero, then no
characters at all are produced.
